# Time to SARS‐CoV‐2 clearance among patients with cancer and COVID‐19

**DOI:** 10.1002/cam4.3708

**Published:** 2021-02-09

**Authors:** Wenxin Xu, Andrew J. Piper‐Vallillo, Poorva Bindal, Jonathan Wischhusen, Jaymin M. Patel, Daniel B. Costa, Mary Linton B. Peters

**Affiliations:** ^1^ Department of Medicine Division of Medical Oncology Beth Israel Deaconess Medical Center Harvard Medical School Boston MA USA; ^2^ Dana‐Farber Cancer Institute Harvard Medical School Boston MA USA

**Keywords:** COVID‐19

## Abstract

**Background:**

For cancer patients, coronavirus disease 19 (COVID‐19) infection can lead to delays in cancer therapy both due to the infection itself and due to the need to minimize exposure to other patients and to staff. Clearance guidelines have been proposed, but expected time to clearance has not been established.

**Methods:**

We identified all patients at a tertiary care hospital cancer center between 25 March 2020 and 6 June 2020 with a positive nasopharyngeal reverse transcriptase polymerase chain reaction (RT‐PCR) test for the severe acute respiratory syndrome coronavirus 2 (SARS‐CoV‐2), a cancer‐related visit within 3 years, and at least one follow‐up assay. We determined the time to clearance using American Society of Clinical Oncology (ASCO), the UK National Institute for Health and Care Excellence (UK‐NICE), and Centers for Disease Control and Prevention (CDC) criteria. A matched non‐cancer comparison cohort was also identified.

**Results:**

Thirty‐two cancer patients were identified. Nineteen were cleared by ASCO criteria, with estimated median time to clearance of 50 days. Fourteen patients resumed chemotherapy prior to clearance. Using UK‐NICE criteria, median time to clearance would have been 31 days, and using CDC criteria, it would have been 13 days. The matched non‐cancer cohort had similar clearance time, but with less frequent testing.

**Conclusion:**

SARS‐CoV‐2 clearance times differ substantially depending on the criteria used and may be prolonged in cancer patients. This could lead to a delay in cancer care, increased use of clearance testing, and extension of infection control precautions.

## Graphical abstract

In this single‐institution retrospective cohort study, the median time to SARS‐CoV‐2 clearance among cancer patients was 50 days using the ASCO/CDC criteria of two negative RT‐PCR assays >24 h apart. Using alternative criteria of one negative RT‐PCR assay (UK‐NICE) or CDC clinical criteria (10 days after first positive RT‐PCR and 3 days after last symptoms), median clearance times were 31 and 13 days, respectively.

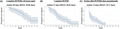

## 1 BACKGROUND

COVID‐19 in oncologic patients presents a dilemma given the competing priorities of providing timely anticancer therapy, avoiding immunosuppression during infection, and minimizing exposure to others. Several different criteria for COVID‐19 clearance have been proposed, but expected time to clearance has not been established.(1, 2, 3)

## 2 METHODS

We identified all patients at a tertiary care hospital between 25 March 2020 and 6 June 2020 with a positive nasopharyngeal SARS‐CoV‐2 RT‐PCR, a cancer‐related visit within 3 years, and at least one follow‐up assay. We selected a comparison cohort of 37 patients without cancer matched for age, gender, race, smoking status, ICU admission at presentation, and COVID‐19‐directed therapy. Data were collected through 30 June 2020. The study was performed as part of an institutional COVID‐19 registry and approved by the institutional review board.

SARS‐CoV‐2 RT‐PCR testing was performed using the Abbott Laboratories m2000 platform with either the Aldatu Biosciences PANDAA qDxTM SARS‐CoV‐2 or Abbott RealTime SARS‐CoV‐2 assays. These assays are widely used in the United States under the FDA Emergency Use Authorization for COVID‐19 testing, and were internally validated at our clinical laboratory and found to have equivalent performance in evaluating known samples. Time to clearance was analyzed using the Kaplan‐Meier probability estimator, for which data were censored at the time of last known assay.

## 3 RESULTS

We identified 32 cancer patients with COVID‐19. Median age was 65, 44% male, and 47% white. One required ICU‐level care at the time of diagnosis, and five were asymptomatic. The majority of these cancer patients had active disease at time of COVID‐19 diagnosis: 16 patients had metastatic disease, 17 were on active treatment, and eight patients were receiving cytotoxic chemotherapy. Four patients had localized disease that had not required cancer‐specific treatment within the past year. Patient and tumor characteristics are described in Table 1.

**TABLE 1:** Baseline patient characteristics

	Cancer patients (n = 32)	Non‐cancer patients (n = 37)
Median age	65	69
Sex		
Male	14	17
Female	18	20
Race		
White	15	17
Black	7	15
Asian	2	0
Other/Hispanic	4	4
Unknown	4	1
Alcohol use		
Yes—Current	8	7
Yes—Past	3	5
No/Unknown	21	25
Tobacco Use		
Yes—Current	2	1
Yes—Past	11	15
No/Unknown	19	21
Cardiac disease (CAD, MI, CHF, or Arrhythmia)		
Yes	10	8
Hypertension		
Yes	16	21
Diabetes mellitus, Type 2		
Yes	7	10
Presenting symptoms		
Symptomatic requiring ICU admission	2	3
Symptomatic not requiring ICU	25	33
Asymptomatic	5	1
ECOG PS		
0–I	24	
II	4	
III	1	
IV	1	
Unknown	2	
Pre‐COVID treatment characteristics		
Received treatment within 6 months of diagnosis	17	
Received chemotherapy	7	
Received immunotherapy	2	
Received targeted therapy	6	
Received hormonal therapy	3	
Tumor type		
Gastrointestinal tumors	9	
Hematologic malignancies	7	
Breast and gynecologic tumors	6	
Genitourinary tumors	5	
Thoracic tumors	3	
Other^a^	2	

^a^ Includes one patient with melanoma and one patient with osteosarcoma.

**FIGURE 1:**
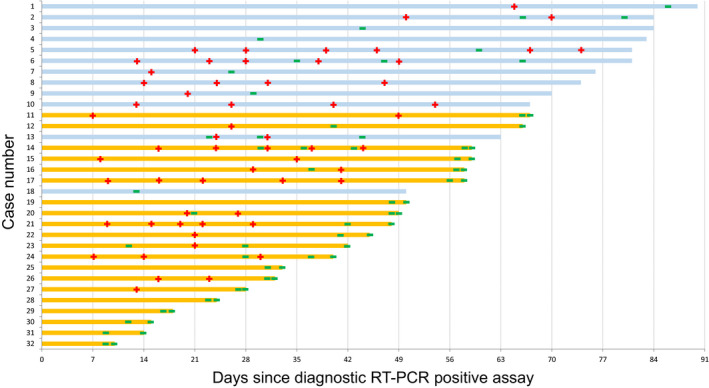
Swimmer's plot of COVID‐19 testing results. (+) indicates SARS‐CoV‐2‐positive RT‐PCR from a nasopharyngeal specimen. (−) indicates SARS‐CoV‐2‐negative RT‐PCR. Orange bars indicate patients who were cleared during the follow‐up period based on two negative RT‐PCR results >24 h apart. Patients #25 and #27 later had a subsequent positive SARS‐CoV‐2 RT‐PCR after meeting clearance criteria

At our institution, and as per current American Society of Clinical Oncology (ASCO) guidelines, discontinuation of COVID‐19 precautions required two consecutive negative PCR tests >24 h apart.(2) Using these institutional criteria, 19 patients were cleared of COVID‐19 precautions, at a median time of 42 days (range 10–67 days; Figure 1). Across the full cohort, median time to clearance was estimated at 50 days (95% CI, 40–66 days). Fourteen patients resumed anticancer treatment prior to clearance. All were asymptomatic from COVID‐19 at the time of resuming treatment, and there were no subsequent COVID‐19 complications. Among the patients who met clearance criteria, two subsequently had a positive assay.

In multivariable Cox models, no significant associations were seen between clearance time and anticancer treatment before or during COVID‐19 positivity or the presence of COVID symptoms. Median Kaplan‐Meier time to two negative RT‐PCR assays was 49 days among the two patients receiving immune checkpoint inhibitors, and 48 days among the seven patients receiving cytotoxic chemotherapy. The number of prior lines of cancer therapy was not a significant predictor for clearance time. As an exploratory analysis, we added age to the multivariable model as either a continuous or a categorical variable (dichotomized at age 65). No association was seen between age and clearance time.

**FIGURE 2:**
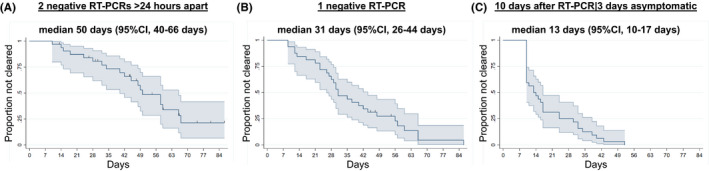
Time to COVID‐19 clearance under different criteria. Kaplan‐Meier probability estimate functions are shown for three alternative criteria for defining COVID‐19 precaution clearance: (A) American Society of Clinical Oncology/Centers for Disease Control and Prevention (CDC) test‐based guideline of two negative RT‐PCR results >24 h apart, (B) UK‐NICE guideline of one negative RT‐PCR after the initial COVID‐19 diagnosis, and (C) revised CDC symptom‐based recommendations for clearance. In panels (A) and (B), patients are censored at the time of last SARS‐CoV‐2 PCR assay. In panel (C), no censoring was needed as all patients achieved clearance at last clinical follow‐up

We calculated COVID‐19 clearance times under alternative criteria (Figure 2). Using United Kingdom‐National Institute for Health and Care Excellence (UK‐NICE) guidelines (one negative RT‐PCR test), median time to clearance would have been 31 days (95% CI, 26–44 days). Using Centers for Disease Control and Prevention (CDC) criteria (10 days after positive test and 3 days after last symptoms), median time to clearance would have been 13 days (95% CI, 10–17 days).

Among the matched non‐cancer cohort, the median time to clearance was 49 days (95% CI, 40 days‐NR) if requiring two negative tests, and 28 days (95% CI, 21–37 days) for one negative test. COVID‐19 RT‐PCR testing was less frequent among non‐cancer patients (mean 3.2 vs. 5.75 tests).

## 4 DISCUSSION

In this study, we observe that time to nasopharyngeal SARS‐CoV‐2 RNA clearance using RT‐PCR in our oncology and non‐oncology patients is longer than the 17–20 days previously reported.(4, 5, 6) As seen with other viruses, an immunocompromised state may be associated with prolonged viral shedding.(7) A recent study showed that patients undergoing CAR‐T cell therapy or stem cell transplantation shed viable SARS‐CoV‐2 for a prolonged period of time, some up to 2 months.(8) In our sample, clearance time was similar in the cancer and non‐cancer cohorts, although direct comparison is difficult as the cancer patients were tested for clearance more frequently due to the urgency of chemotherapy treatment. Clearance time did not correlate with administration of cytotoxic chemotherapy and presumed immune compromise.

At our institution, patients are not able to return to the cancer center for appointments or treatment until they are cleared by two negative PCR assays. In the interim, patients can be seen in a dedicated COVID‐19 assessment and treatment unit. Despite opening this unit, access to care and chemotherapy treatment is more limited for these patients. In addition, this dedicated unit requires allocation of staff and personal protective equipment, both relatively constrained resources. Clearance testing requires repeated visits over multiple weeks, with some patients receiving up to 10 tests; this is burdensome to the patient and caregivers, as well as using testing resources. The dedication of testing and treatment resources is important and necessary, but understanding the true need for a COVID‐19 dedicated cancer treatment plan will be important as we move into the next phases of the pandemic.

Of note, on 22 July, CDC again revised guidelines for clearance of COVID‐19‐positive patients, suggesting that a purely time‐based strategy allowing 10 days from symptom onset would be sufficient for most patients, barring severe infection or severe immunocompromised state.(9) Current CDC guidance includes all cancer patients in the immunocompromised category, and patients receiving chemotherapy are considered severely immunocompromised.(1, 10) Current CDC guidelines, therefore, support the use of test‐based criteria for patients receiving cytotoxic chemotherapy. However, not all patients receiving chemotherapy are equivalently immunocompromised, and our data show that using clinical clearance criteria would further shorten the clearance period for most patients. There are, however, limited data on this population of patients on active immune suppression.

In addition, further elucidation of the relationship between viral shedding and infectivity is needed to guide the development of future guidelines. Recent data have shown that PCR positivity does not correlate with the ability to culture viable virus, and that only rarely is viable virus isolated from nasal secretions more than 10 days after infection.(11, 12) Positive PCR testing may not be informative more than 10 days after infection.

COVID‐19 has caused substantial global delays in cancer care, including delays in cancer screening as well as an anticipated increase in cancer‐related deaths even among patients who did not develop COVID‐19 infection.(11, 12, 13, 14) Optimization of precautions and clearance guidelines is therefore essential for improving the care of cancer patients both with and without COVID‐19.

## 5 CONCLUSIONS

At our cancer center, patients with cancer who developed COVID‐19 infection had prolonged PCR positivity, weeks after symptom onset. Choosing criteria for clearance in cancer patients must take into account the tradeoffs between the increased risk of poor clinical outcomes from COVID‐19, the clinical risk from delays in anticancer therapy, and the risk of exposing others. In our cancer center, the difference between using ASCO guidelines versus CDC guidelines leads to a difference of more than a month, during which time these patients are restricted from access to the regular cancer center resources. Symptom/time‐based clearance strategies may result in shorter times to discontinuation of precautions, though the risks of earlier return to routine cancer care remain unclear.

## CONFLICT OF INTEREST

JMP: Received honoraria from Radius, research grant support from Breast Cancer Research Foundation, institutional research support from Genentech, Sanofi, Odonate Therapeutics, all outside submitted work. DBC: Reports personal fees (consulting fees and honoraria) and non‐financial support (institutional research support) from Takeda/Millennium Pharmaceuticals, AstraZeneca, Pfizer, and personal fees (consulting fees) from Blueprint Medicines, as well as non‐financial support (institutional research support) from Merck Sharp and Dohme Corporation, Merrimack Pharmaceuticals, Bristol‐Myers Squibb, Clovis Oncology, Spectrum Pharmaceuticals and Tesaro; all outside the submitted work. MLBP: Received honoraria from Bayer, Exelixis, and Agios, travel support from Halozyme, AstraZeneca, and Exelixis, research support from Bayer, institutional research support from Taiho, AstraZeneca, BeiGene, Berg, Merck, all outside the submitted work. Other authors have no potential conflicts.

## AUTHOR CONTRIBUTION

Authors WX and MLBP had full access to all the data in the study and take responsibility for the integrity of the data and the accuracy of the data analysis.

## Data Availability

The data that support the findings of this study are available on request from the corresponding author. The data are not publicly available due to privacy or ethical restrictions.
